# Hamman’s Syndrome after Vaginal Delivery: A Case of Postpartum Spontaneous Pneumomediastinum with Subcutaneous Emphysema and Review of the Literature

**DOI:** 10.3390/healthcare12131332

**Published:** 2024-07-03

**Authors:** Kristina Olafsen-Bårnes, Marte Mari Kaland, Karol Kajo, Lars Jakob Rydsaa, Jozef Visnovsky, Pavol Zubor

**Affiliations:** 1 Department of Obstetrics and Gynecology, Helgeland Hospital, 8802 Sandnessjøen, Norway; kristina.benedikte.olafsen@helgelandssykehuset.no (K.O.-B.); marte.mari.nordeide.kaland@unn.no (M.M.K.); 2 Department of Pathology, St. Elisabeth Cancer Institute, 81250 Bratislava, Slovakia; karol.kajo1@gmail.com; 3 Department of Radiology, Helgeland Hospital, 8802 Sandnessjøen, Norway; lars.jakob.rydsaa@helgelandssykehuset.no; 4 Faculty of Health Care, Catholic University, 03401 Ruzomberok, Slovakia; jozo.visnovsky@gmail.com; 5 VISNOVSKI Ltd., 03601 Martin, Slovakia; 6 OBGY Health & Care Ltd., 01001 Zilina, Slovakia; 7 Department of Obstetrics and Gynecology, Nordland Hospital, 8450 Stokmarknes, Norway

**Keywords:** delivery, Hamman’s syndrome, pneumomediastinum, pregnancy

## Abstract

Hamman’s syndrome is a rare condition that mostly affects young males, often with a predisposition to asthma. It includes the presence of free air in the mediastinum and subcutaneous emphysema with no other underlying cause such as trauma, infection, or administration of any sort of mask support with hyperpressure. It occurs spontaneously and often in association with a prolonged Valsalva maneuver. This might explain why there are some cases of Hamman’s syndrome among young females giving birth. Here, we present a case report of a 24-year-old non-smoker primigravida with Hamman’s syndrome. She presented with symptoms a few hours after an uncomplicated vaginal delivery at 40 + 1 weeks of pregnancy where the active phase of labor lasted for three hours with normal progress. The second stage lasted for 30 min, with no signs of distress on CTG. The symptoms (pain in the right ear, swelling and pain in the neck, chest tightness, shortness of breath, dysphagia, odynophagia, and pain in the upper thorax on the right side) and objective findings as subcutaneous crepitations in the neck, parasternal region, right axillary fossa, clavicle and over the chest resolved spontaneously after a few days of observation and conservative management. We also give a systemic review of reported cases since 2000 to provide an overview of the pathomechanism, symptoms, diagnostics, treatment, and management of this condition. Hamman’s syndrome is a rare, usually benign, but potentially serious complication that can occur during the second stage of labor. Diagnostics include inquiring about typical symptoms, clinical examination, and chest x-ray or CT scan. Treatment is usually conservative with oxygen, bronchodilators, and pain relief. The recurrence rate is low and there is no contraindication to vaginal delivery in future pregnancies. However, it is suggested that physicians and midwives be cautious and consider a low threshold for instrumental delivery or cesarean section to avoid excessive Valsalva maneuvers.

## 1. Introduction

Spontaneous pneumomediastinum (SPM) during labor is a rare event, occurring in about 1 in 100,000 deliveries. Together with subcutaneous emphysema, it is called Hamman’s syndrome [1]. The condition was named after Louis Hamman (1877–1946), the physician who described it in several case reports from 1939 to 1945 in postpartum women [2,3].

SPM is defined as the presence of free air in the mediastinum with no underlying trauma and mostly affects young males and pregnant females. It may be associated with a pulse-synchronous crunching sound, referred to as the “Hamman’s sign”, best heard when the patient is lying in the left lateral decubitus position [4,5].

Hamman’s syndrome may occur during prolonged labor, usually in the second stage, after a prolonged Valsalva maneuver. Here, it is pneumomediastinum and subcutaneous emphysema not linked to any sort of mask support with hyperpressure. Other predisposing events may be intensive coughing, retching/vomiting, or physical activity [6].

The condition is usually benign and self-limiting, but in rare cases, there may be complications such as significant dyspnea and chest pain, and even the development of malignant pneumomediastinum, which requires surgical intervention [7]. In this paper, we report a case of Hamman’s syndrome in a 24-year-old primigravida with underlying asthma. We examine the pathomechanism and management of this condition, and provide an overview of 42 other published cases in the last two decades.

## 2. Case Presentation

A 24-year-old primiparous woman, with a normal pregnancy, presented to the maternity ward in spontaneous labor, with regular uterine contractions at 40 + 1 weeks. She was a non-smoker and denied drinking alcohol or using illicit drugs. Her body mass index was 27.1, and her past medical history was significant for depression, ADHD (Attention Deficit Hyperactivity Disorder), and childhood asthma, without flare-ups or need for medical treatment in the last years. There was no history of any heart condition.

**Figure 1:**
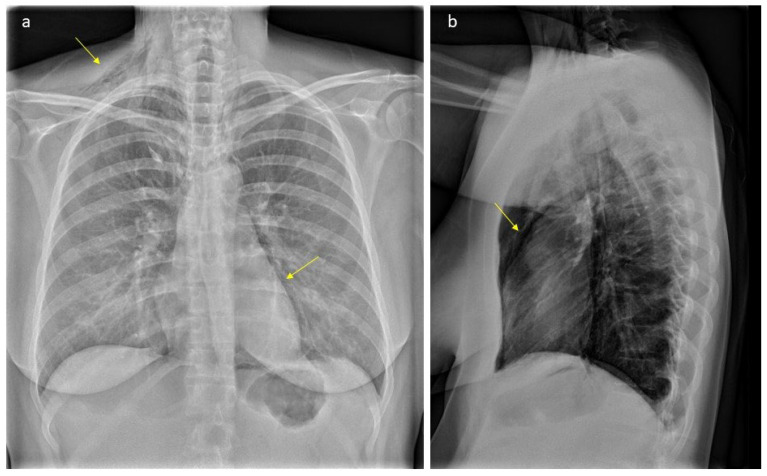
Plain radiograph scan showing typical gas accumulations seen subcutaneously and with pneumomediastinum (arrows, part (**a**)): There is significant subcutaneous emphysema, more pronounced on the right side (arrow, part (**a**)). We can see an outlining of the pericardium both in lateral projection (arrow, part (**b**)) and with a “continuous diaphragm sign” on frontal projection (arrow, part (**a**)). You can also see continuous lucencies along upper mediastinum to the neck, through upper thoracic aperture.

**Figure 2:**
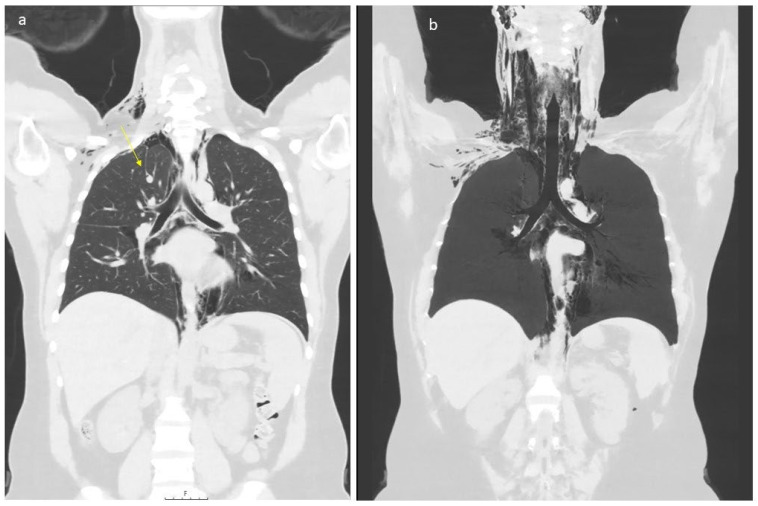
Low dose CT scan showing air within the mediastinum starting caudally in the level of hiatus aorticus, surrounding the pericardium, throughout the mediastinum. At the neck level, air was seen subcutaneously (part (**a**)), along the great vessels (carotid space), and in the retropharyngeal space (part (**b**)). The patient has an “azygos lobe”, a normal variant with the vena azygos running laterally with a pleural fissure surrounding it (arrow, part (**a**)). Air bubbles can be seen along the vein and small amounts of air within the pleural cavity apically on the right side. Apart from this, no signs of pneumothorax. Small amounts of pleural effusion are seen bilaterally. The upper abdomen was included in the low-dose scan, without any signs of air below the diaphragm.

In the latent phase of delivery, she received morphine for analgesia. The water broke spontaneously, and the amniotic fluid was discolored. The patient had a normal temperature and there was no fetal tachycardia or other signs of fetal distress on cardiotocography (CTG). The active phase of labor lasted for three hours with normal progress. After one hour of passive descent, she started pushing, and the second stage lasted for 30 min. It was a spontaneous vaginal delivery of a healthy baby with a normal Apgar score (9-10-10) weighing 4170 g. There was a normal expulsion of the placenta, and there was normal bleeding. Eight hours after delivery, she complained of pain in the right ear, swelling and pain in the neck, chest tightness, shortness of breath, dysphagia, odynophagia, and pain in the upper thorax on the right side. Her vital signs were stable (BP 128/67 mmHg, pulse 91/min, temperature 36.0 °C, respiratory rate 14/min, and oxygen saturation 99% on room air). The ECG was unremarkable. Blood gas showed normal values with pH 7.45 and pCO_2_ 4.2 kPa. A PCR test from the nasopharynx was negative for viral infections (analyzed for Adenovirus DNA, Chlamydia pneumoniae DNA, Coronavirus 229E RNA, Coronavirus NL63 RNA, Coronavirus OC43 RNA, SARS-CoV-2 RNA, Enterovirus RNA, Influenza A virus RNA, Influenza B virus RNA, Humant metapneumovirus RNA, Mycoplasma pneumoniae DNA, Humant parainfluenza virus 1 RNA, Humant parainfluenza virus 2 RNA, Humant parainfluenza virus 3 RNA, Humant parainfluenza virus 4 RNA, Bordetella pertussis DNA, Bordetella parapertussis DNA, Rhinovirus RNA, and Respiratorisk syncytial virus RNA). There were no findings on otoscopy. On palpation, there were subcutaneous crepitations in the neck, parasternal region, right axillary fossa, clavicle, and over the chest. Auscultation of the heart and lungs was normal. A chest X-ray (Figure 1) was taken immediately, revealing subcutaneous emphysema extending bilaterally to the neck but more prominent on the right side, and suspicious for pneumomediastinum. There were no signs of pneumothorax, esophageal, or skeletal pathology. The heart configuration was normal. The patient received 1 g of paracetamol and 2.5 mg of morphine intravenously. The situation was clinically stable. The next day, a CT scan (Figure 2) without contrast was performed, confirming pneumomediastinum, with air extending from the diaphragm up to the thoracic apexes and across the larynx. There were discreet amounts of pleural fluid bilaterally, and subcutaneous emphysema from the base of the skull to the neck and upper thorax.

The patient was reviewed by the medical team (radiologist, gynecologist, and surgeon) and diagnosed with Hamman’s syndrome. As the patient was hemodynamically stable, she was managed conservatively with observation and analgesics. Her symptoms resolved gradually over the next three days and she was discharged home on her third day postpartum. She was advised to avoid strenuous physical activity for the next four weeks. At the 6-week postnatal follow-up, she was well and completely without symptoms. Follow-up correspondence was also conducted over the phone at five months. The patient had recovered well and was now practicing normal physical activity. 

## 3. Discussion

Hamman’s syndrome is a rare clinical entity. Its incidence is 1:100,000 women giving birth. The incidence is higher in cases of accidents or emergencies and has a male predisposition, accounting for 76% of cases [8]. It is believed to be a result of a sudden increase in intra-alveolar pressure. Mostly it is associated with the Valsalva maneuver, extensive vomiting, or coughing, all of which can occur in pregnancy and labor.

There have been several cases of Hamman’s syndrome occurring in labor, but it has also been reported in association with other medical conditions, such as diabetic ketoacidosis with repeated vomiting or Kussmaul breathing [9,10] and bronchial asthma, with vomiting and coughing as common precipitating factors [11,12]. There have also been reports of SPM occurring after intense coughing during strenuous physical activity [13] or hyperemesis gravidarum [14].

In our case, we believe the extensive breathing during the first stage and intensive Valsalva maneuver in the second stage of labor, in a patient with underlying bronchial asthma, led to the development of symptoms of Hamman’s syndrome after delivery. Moreover, it is known that a history of ADHD and anxiety could be another favorable factor that would cause hyperventilation during delivery, thus this underlying condition could impact the development of spontaneous pneumomediastinum as well. A similar situation in a woman with a history of bipolar disorder and anxiety was reported previously [15].

The CT scan of the chest was taken to exclude other severe diseases like pulmonary embolism, amniotic fluid embolism, myocardial infarction, and Boerhaave syndrome.

The pathophysiology of Hamman’s syndrome is explained as follows: The intra-alveolar pressure is acutely increased during the Valsalva maneuver, causing rupture of marginal alveoli adjacent to blood vessels. The free air moves from ruptured alveoli along peribronchial vascular sheaths towards the hilum of the lung. From there, it extends proximally and can spread within the mediastinum, pericardium, neck, subcutaneous tissue, and retroperitoneum. The absence of transverse fascial planes in the mediastinum allows the unobstructed passage of air along tissue planes into the neck and around the larynx. The air may also be trapped between the parietal and visceral pleura, causing pneumothorax. The pressure of the interstitial air rarely causes respiratory compromise [1]. Coughing, vomiting, screaming, and the force of pushing in labor, together, can increase intrathoracic pressure.

The most common symptoms are chest (retrosternal) pain radiating to the back or neck, dyspnea, and swelling of the face and neck. The crepitus palpable in the face and neck is pathognomonic of the condition [4,16]. Other symptoms include change of voice (dysphonia), cough, sore throat, tachycardia, dysphagia, and hemoptysis. A characteristic sign is the bubbling or crunching sounds over the heart, synchronous with the cardiac cycle, known as Hamman’s sign or murmur. The occurrence of the symptoms, time onset after/during delivery, severity of the condition, and management can be very variable, as described in our summarized overview of the reported cases over the last two decades (Table 1).

In our case, the woman presented immediately after delivery with pain around the ear and the feeling of a plugged ear. A few hours later she reported swelling of the neck, chest tightness, and shortness of breath. On examination, crepitus on the neck and thorax was obvious, but there was no typical Hamman’s murmur.

Hamman’s syndrome is usually a benign and non-recurrent condition. However, in rare cases, it may be life-threatening and lead to cardiac tamponade with significant hemodynamic compromise. Such situations require surgical intervention [17]. Prior to any intervention, it is important to exclude other serious, potentially life-threatening conditions such as esophageal rupture (cancer-related), Boerhaave syndrome (rupture of the esophagus due to forceful vomiting), pharyngeal rupture, pulmonary embolism, amniotic fluid embolism, aortic dissection, myocardial infarction, pneumopericardium, or pneumothorax of any cause [18,19].

Apart from the clinical picture, a CT scan is the gold standard in diagnosing pneumomediastinum. In a systematic review, it was found that about 30% of cases of pneumomediastinum were poorly detected by chest X-ray, but were easily detected on a CT scan [20]. The CT scan also provides more accurate information on the extension of subcutaneous emphysema and other thoracic pathologies. An additional tool that can be considered is diagnostic endoscopy (bronchoscopy or esophagoscopy) or esophagography [21]. While the direct relevance of these methods for the detection of Hamman’s syndrome is lacking, they can exclude serious injuries (ruptures) of the airways or upper gastrointestinal tract leading to the pneumomediastinum.

Initial management is supportive treatment with oxygen, sedatives, and analgesics, as needed. In severe cases, treatment with antibiotics and bronchodilators, along with oxygen support, may be added. The patient should be reassured about a good prognosis and expected spontaneous resolution within 3–14 days [22]. Patients can be discharged if they are in good general condition and do not have a significant pneumothorax. There is no recommended routine follow-up.

There have been a few reports of cases with spontaneous pneumomediastinum occurring in the setting of hyperemesis gravidarum in early pregnancy [14] or spontaneously in the third trimester. Here, the operative delivery should be considered to prevent the worsening/recurrence of this condition [23].

**Table 1:** An overview of 42 previously published cases of pregnancy associated with Hamman’s syndrome (database Pubmed.gov from 2000 to 3/2024).

Author	Age y/o	Parity	When Symptoms Developed	Duration of Labor	Week of Gestation	Treatment
Sutherland et al. 2002 [24]	32	Para 1	Postpartum	8 h	N/A	None
Sutherland et al. 2002 [24]	22	Para 1	13 h postpartum	N/A	N/A	None
Miguil et al. 2004 [25]	19	Para 0	N/A	N/A	40	Oxygen and analgesics, C-section
Duffy 2004 [26]	19	Para 0	2 h	90 min 2nd stage	40	Oxygen v analgesics
Bonin et al. 2006 [27]	27	Para 0	2nd stage	6 h	38	Lorazepam for anxiety and anxiolytics for dyspnea
Norzilawati et al. 2007 [28]	21	Para 0	12 h postpartum	4 h, 100 min 2nd stage	40	None
Yadav et al. 2008 [29]	21	Para 0	2nd stage	2nd stage 1.5 h	N/A	Oxygen and analgesics
Mahboob et al. 2008 [30]	24	Para 0	18 h postpartum	N/A Normal	39	Oral antibiotics, IV fluids, and analgesics
Zapardiel et al. 2009 [31]	29	Para 0	Postpartum	N/A	39	Oxygen
Revicky et al. 2010 [32]	32	Para 0	3 h	14 h	40	None
Beynon et al. 2011 [33]	18	Para 0	8 h postpartum	4 h	39	Antibiotics and analgesics
Wozniak et al. 2011 [34]	20	Para 0	5 h postpartum	9 h	41	Observation
Shrestha et al. 2011 [35]	19	Para 0	N/A	N/A	36	None
Kuruba et al. 2011 [1]	32	Para 1	2nd stage	1.5 h	40	None
McGregor et al. 2011 [36]	27	Para 0	2nd stage	7.5 h	40	Oxygen and analgesics
Houari et al. 2012 [37]	21	Para 0	Postpartum	N/A	40	Conservative management
Kandiah et al. 2013 [38]	25	Para 0	2nd day postpartum	2nd stage 3 h, 16 min. Ending in a C-section	40	Observation
Kandiah et al. 2013 [38]	30	Para 0	2nd stage	6 h	38	Observation
Kouki et al. 2013 [39]	23	Para 0	2nd stage	9 h	40	Oxygen and analgesics and sedatives
Khoo et al. 2015 [40]	33	Para 0	2nd stage	12 h	40	Analgesics and bed rest
Cho et al. 2015 [7]	28	Para 0	2nd stage	5 h	36	Oxygen and analgesics
Wijesuriya et al. 2015 [41]	24	Para 0	N/A	N/A	N/A	N/A
Khurram et al. 2015 [4]	24	Para 1	2 h postpartum	2nd stage prolonged	40	None
Scala et al. 2016 [42]	30	N/A	2nd stage	N/A	40	None
Elshirif et al. 2016 [43]	27	Para 0	4 h postpartum	19 h 2nd stage 3 h	41	Analgesics, oxygen, and antibiotics
Berdai et al. 2017 [44]	22	Para 0	2nd stage	2 h	40	Oxygen
Lou et al. 2017 [45]	29	Para 0	2nd stage	Prolonged	At term	Supportive
Sagar et al. 2018 [46]	22	Para 0	3 h postpartum	4.5 h	37	None
Khan et al. 2018 [47]	30	Para 0	N/A	N/A	N/A	Antibiotics, oxygen, and bronchodilators
Jakes et al. 2019 [48]	23	Para 0	40 min postpartum	2nd stage 2 h	38	Oxygen
Madhok et al. 2019 [49]	21	Para 0	2 h postpartum	3 h	39	None
Lee et al. 2019 [50]	31	Para 0	2nd stage	8.4 h	41	IV antibiotics, hydrocortisone and Loratadine
Chavan et al. 2019 [51]	33	Para 0	10 h postpartum	90 min 2nd stage	38	Oxygen and analgesics
Opstelten et al. 2019 [52]	25	Para 0	2nd stage	N/A	N/A	N/A
Oshovskyy et al. 2020 [53]	34	Para 4	2nd stage	4.5 h	39	Pigtail catheter
Badran et al. 2020 [54]	N/A	Para 0	4 h postpartum	N/A	Full term	Nil by mouth
Zethner-Møller et al. 2021 [55]	35	Para 1	2nd stage	N/A	36	Oxygen
Mullins et al. 2021 [56]	17	Para 0	postpartum, prolonged second stage	N/A	39	Oxygen and opioids
La Verde et al. 2022 [18]	23	Para 0	2nd stage	5 h	41	None
Gomes et al. 2022 [6]	21	Para 0	2nd stage	N/A	40	C-section and observation
Peña-Vega 2023 [57]	18	Para 0	30 h postpartum	12 h	39	Oxygen
Chooi et al. 2023 [58]	22	Para 0	2nd stage	3 h 2nd stage	39	None
Hülsemann et al. 2023 [59]	21	Para 0	2nd stage	Prolonged	N/A	N/A
Inesse et al. 2023 [60]	29	Para 0	1 h postpartum	2nd stage lasted 2 h, 40 min active pushing	40	None
Chen et al. 2023 [61]	20	Para 0	Immediately after delivery	Prolonged	43	Analgesics and antibiotics iv

Abbreviations: N/A—not available; IV—intravenous; h—hours.

## 4. Conclusions

Postpartum pneumomediastinum (Hamman’s syndrome) is a rare complication, and its timely diagnosis is necessary for patient safety and management. Most cases in pregnant women occur in the second stage of labor, as a result of excessive straining and Valsalva maneuver. The recurrence rate in subsequent pregnancies is low, and there are no established guidelines on the management of this condition. It is suggested that measures that can be implemented should aim to minimize barotrauma with the low threshold for instrumental or operative delivery and limit the duration of the second stage of labor. However, this is not evidence-based and may require a meta-analytical approach. Furthermore, we need to pay more attention to the predisposing risk factors for this condition (e.g., asthma, smoking, emphysema, chronic cough conditions, chronic lung obstructive diseases, history of pneumothorax, diabetes, or hyperemesis conditions). This will enhance the practical management of pregnant women, especially if we could develop a model for risk identification and stratification focusing on identifying a subpopulation of pregnant women at high risk for Hamman’s syndrome. This stratification could be crucial for improving patient outcomes and minimizing the incidence of this rare syndrome.

## Data Availability

Data sharing is not applicable to this article as no datasets were generated or analyzed during the current study. The data presented in this study are available on request.
